# Whole-Body Roll Tilt Influences Goal-Directed Upper Limb Movements through the Perceptual Tilt of Egocentric Reference Frame

**DOI:** 10.3389/fpsyg.2018.00084

**Published:** 2018-02-15

**Authors:** Keisuke Tani, Yoshihide Shiraki, Shinji Yamamoto, Yasushi Kodaka, Keisuke Kushiro

**Affiliations:** ^1^Graduate School of Human and Environmental Studies, Kyoto University, Kyoto, Japan; ^2^The Japan Society for the Promotion of Science, Tokyo, Japan; ^3^Faculty of Sport Sciences, Nihon Fukushi University, Aichi, Japan; ^4^National Institute of Advanced Industrial Science and Technology, Neuroscience Research Institute, Ibaraki, Japan

**Keywords:** gravitational direction, goal-directed upper limb movement, whole-body tilt in roll plane, body longitudinal axis, egocentric reference frame

## Abstract

In our day-to-day life, we can accurately reach for an object in our gravitational environment without any effort. This can be achieved even when the body is tilted relative to gravity. This is accomplished by the central nervous system (CNS) compensation for gravitational forces and torque acting on the upper limbs, based on the magnitude of body tilt. The present study investigated how performance of upper limb movements was influenced by the alteration of body orientation relative to gravity. We observed the spatial trajectory of the index finger while the upper limb reached for a memorized target with the body tilted in roll plane. Results showed that the terminal location of the fingertip shifted toward the direction of body tilt away from the actual target location. The subsequent experiment examined if the perceived direction of the body longitudinal axis shifted relative to the true direction in roll plane. The results showed that the perceived direction of the body longitudinal axis shifted toward the direction of the body tilt, which correlated with the shift of the terminal location in the first experiment. These results suggest that the dissociation between the egocentric and gravitational coordinates induced by whole-body tilt leads to systematic shifts of the egocentric reference frame for action, which in turn influences the motor performance of goal-directed upper limb movements.

## Introduction

We can accurately move the upper limb without any effort in spite of our body being affected by gravity. This is accomplished by the CNS estimating and compensating the effect of gravity preceding, and/or during the movement. Several previous studies have elucidated how the CNS compensates for external forces applied to the upper limbs, by observing behavior and kinematics when an external force is experimentally altered within the force field (Shadmehr and Mussa-Ivaldi, 1994; Sainburg et al., 1999; Scheidt et al., 2005), and during the parabolic flight (Fisk et al., 1993; Papaxanthis et al., 2005; Crevecoeur et al., 2009; Gaveau et al., 2016). Additionally, body orientation changes, relative to gravity also induce alteration of the forces (i.e., gravitational force) acting on the upper limb. Although a few studies have investigated how the body orientation changes due to whole-body tilt affect the performance of upper limb reaching movements (Smetanin and Popov, 1997; Bourdin et al., 2001; Prieur et al., 2006), the functional relationship between gravitational direction relative to the body and its effect on upper limb movements is largely unknown.

Multisensory inputs such as visual, somatosensory, and vestibular contribute for the CNS to accurately estimate gravitational direction (Bisdorff et al., 1996; Bronstein, 1999). If vision is not available, estimation of the gravitational direction is best achieved when it is aligned with the direction of the body longitudinal axis, whereas the estimation error increases as their directions are dissociated in space by whole-body tilt. It is known that the estimation error develops systematically depending on the amount of body tilt during the subjective visual vertical (SVV) task, wherein the subjects are instructed to adjust a visual rod to the perceived vertical (for review, Carriot et al., 2008). For a relatively small angle (<60°) of body tilt, the perceived vertical tends to shift opposite to the direction of body tilt, i.e., there is overestimation of the body tilt (Day and Wade, 1969; Ebenholtz, 1970; Van Beuzekom and Van Gisbergen, 2000; Tarnutzer et al., 2010), which is referred to as the “E-effect” (Muller, 1916). These findings lead us to imagine that the error in perception of the gravitational direction during whole-body tilt would, in turn, cause spatially inaccurate motor planning by the CNS. Consequently, it is assumed that when the body is tilted in roll plane with a small angle of tilt, the upper limb movements are shifted opposite to the direction of the body tilt, as they reflect the perceived direction of gravity.

In this study, we investigated how the performance of upper limb movements was modulated at a small angle of whole-body tilt (<60°) in roll plane. In addition, modulation of motor performance, if any, is related to the spatial property of the perceived egocentric space during whole-body tilt in roll plane. A memory-guided reaching task along the longitudinal axis of the body was performed for testing the motor performance (Experiment 1), followed by testing of the perceived body longitudinal axis to evaluate perception of the egocentric space (Experiment 2). Finally, we demonstrated alteration of the upper limb movement at small angles (8° and 16°) of body tilt in roll plane, which was correlated and potentially explained by the property of the perceived egocentric space.

## Experiment 1

### Methods and Materials

#### Subjects

Fourteen healthy male subjects (aged 21–25 years) participated in this study. All subjects were right-handed with normal vision and did not have any neurological, muscular, or cognitive disorder. Handedness was determined by means of Edinburg Handedness Inventory (Oldfield, 1971). All participants gave written informed consent prior to this study. This study was approved by the Ethics Committee of the Graduate School of Human and Environmental Studies, Kyoto University and was conducted in accordance with the Declaration of Helsinki.

#### Apparatus

**FIGURE 1:**
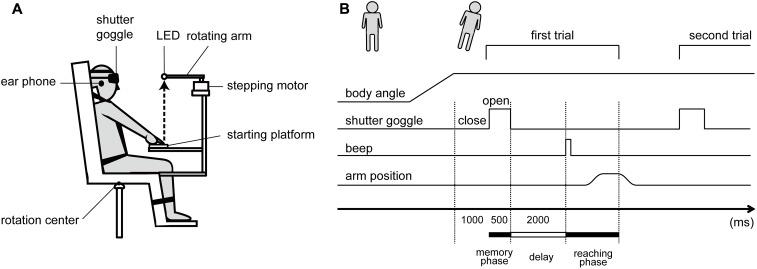
**(A)** Schematic diagram of the experimental setup. **(B)** Schematic diagram of the experimental procedure.

The subjects were comfortably seated on a racing car seat (RECARO SR-7 KK100, RECARO Japan, Japan) mounted on a custom-made tilt-table in a completely darkened room (**Figure 1A** for the schema). The trunk was firmly secured to the seat with a four-point safety belt in natural position. The head was restrained to the seat in straight-ahead position tightened by a Velcro band horizontally placed on the forehead. The legs were restrained to the footrest in comfortable position with another band. The axes underneath the tilt-table were lengthened or shortened by the servo motors, so that the seat could be tilted in roll plane around a rotation center underneath the center of the tilt-table. The velocity of the tilt was set at 2.75°/s, following the initial acceleration phase at 0.58°/s^2^. Induced acceleration due to the tilt motion seems to be higher than the detection threshold of the semicircular canals (SCC, 0.05°/s^2^, Diamond and Markham, 1983), which could potentially affect perception of the verticality (Jaggi-Schwarz and Hess, 2003) and arm movements (Bockisch and Haslwanter, 2007). However, in a preliminary experiment, we confirmed that the tilt motion in roll plane with the parameters in our setup induced no visible nystagmus after arriving at the tilted position. Therefore, the dynamic effects arisen by the tilt motion were assumed to be negligible in the present study.

A light-emitting diode (LED) as the target for the memory-guided reaching was placed in front of the center of the eyes. The LED distance from the head was individually adjusted to the upper limb length. In order not to provide subjects tactile feedback information regarding the target location, which would arise when contacting with the LED, the rotation arm (18 cm in length) with LED was horizontally turned by a stepping motor (TS3103N124, TAMAGAWA SEIKI, Japan) when the reaching task was performed. As a result, the subjects pointed on the space. A platform made of squared wood (8 × 13 cm) was placed underneath the target to define the starting position of the reaching movement. A press button embedded in that starting platform, defined as the starting position of the index finger, was aligned to the location 43.0 cm underneath the target. All of the apparatus mentioned above were firmly fixed on the tilt-table with the ridged metal frame system (Green Frame, SUS, Japan), which enabled the stabilization of the spatial property of the reaching task in the body/head-centric coordinate system even when the whole apparatus on the tilt-table was tilted.

The subjects wore the custom-made head-mount shutter goggle controlled by the microcomputer (Arduino UNO, Arduino SRL, United States), which could restrict the vision in temporal. In addition, the subjects were provided with white noise via the earphones to prevent any spatial cues due to the noise from the surrounding environment.

An infrared reflective marker (3 mm in diameter) was placed on the center of subjects’ right index fingernail. The three-dimensional trajectory of the index finger during the reaching task and the spatial location of the target were recorded with the motion capture system (OptiTrack, NaturalPoint, United States) at a 100-Hz sampling rate.

#### Procedures

At the beginning of each trial, the subjects sat on the upright seat with the shutter goggle closed, placed the right hand on the starting platform with the upper limb fully extended, and maintained the index finger on the press button. As the experimenter announced the start of the trial, the tilt-table was slowly tilted right- or leftward in roll plane. Five whole-body tilt conditions in roll plane were applied: 0°, ±8°, and ±16°, with positive and negative values for right side down (RSD) and left side down (LSD) tilt, respectively. One second after a certain tilted position was achieved, the shutter opened and the LED as the target became visible only for 500 ms (memory phase in **Figure 1B**). Subsequently, the shutter closed again, and the stepping motor rotated 90° clockwise; hence, the LED slipped off from the spatial area where the subjects’ finger could have arrived. After a 2-s delay period, a single beep sound was presented to the subjects via the earphones, prompting the subjects to reach for the memorized target location (reaching phase). The subjects were instructed to reach the right fingertip upward toward the memorized target with the arm kept extended, under the instruction “perform as rapidly and accurately as possible,” and to maintain the final reaching location for approximately 1 s, which terminated the trial. After the trial, the subjects returned the hand to the starting position, relaxed, and prepared for the next trial, while the target was rotated back to the initial location. One trial lasted <8000 ms and was successively repeated 14 times at the identical tilted position, which consisted one block, and then the subject was returned to the initial upright position.

Each subject performed five blocks with 5-min rests in between, with a total of 70 trials. The order of the blocks to proceed was initiated by the upright (0°) condition, and then the other tilt conditions were followed by the randomized order. During the rest period, the room lights were turned on with the shutters of the goggles opened to prevent dark adaptation and the subjects were encouraged to relax to avoid fatigue.

#### Data Analysis

Three-dimensional locations of the right index finger and target LED obtained by the motion capture system were initially filtered with a second-order Butterworth low-pass filter, with 10-Hz cut-off frequency. The present study focused on and required only two-dimensional data in roll plane. The horizontal and vertical axes in earth-fixed coordinate system were defined as X and Y, with rightward and upward as positive values, respectively. The recorded data in the earth-fixed coordinate system was converted by the rotation matrix with the tilt angle so that the spatial relationship between the starting position and the target was identical throughout all the conditions, in order to compare the accuracy and precision of reaching performance across all conditions (see next paragraph). Specifically, the converted data (X_b_, Y_b_) in the body-fixed (egocentric) coordinate system spatially aligned across the conditions was calculated from the finger location data (X, Y) at the tilt angle (α) around the starting finger location (x, y) across the tilt conditions with the following formula:

$$\begin{array}{c} {\text{X}}_{\text{b}}=(\text{X}-\text{x})\times \text{cos}(\text{α})-(\text{Y}-\text{y})\times \text{sin}(\text{α})\\ {\text{Y}}_{\text{b}}=(\text{X}-\text{x})\times \text{sin}(\text{α})+(\text{Y}-\text{y})\times \text{cos}(\text{α}) \end{array}$$

Consequently, X_b_ and Y_b_ values were expressed in the spatially aligned coordinate system, but with the origins deviated due to the different rotation centers between the above calculation (starting position) and the actual rotation center of the tilt-table. Despite that, the conversion aligned the spatial direction between the starting and target positions, and enabled the direct comparison of the reaching performances across all tilt conditions.

For each trial, the movement onset and offset were defined as the first time the tangential velocity was more than 5% of peak velocity and less than that after movement onset. To assess the influence of body-tilt on reaching performance, we firstly calculated the constant error in degrees, indicating the error from the target with positive/negative sign. For inducing constant error, we calculated two parameters: (1) initial direction error (IDE), the angular difference between the vectors from the starting position to the target and to the fingertip location when peak acceleration appeared, and (2) final direction error (FDE), the angular difference between the vectors originating from the starting position to the target and to the location where the fingertip finally arrived. The positive and negative values correspond to the clockwise (rightward) and counterclockwise (leftward) direction in roll plane from the subjects’ view, respectively. Subsequently, as the parameter for accuracy of reaching performance, constant errors of IDE and FDE were averaged for each subject at each tilted position. As the parameter for precision of reaching movements, intra- (within-) subject variabilities of IDE and FDE were calculated by averaging the standard deviation (SD) of IDE and FDE within subjects at each tilt position.

Because the previous studies (Wade, 1970; Tarnutzer et al., 2013) have shown that sustained static whole-body tilt in roll plane induced an alteration of verticality perception, the reaching performances in this study might gradually change as the duration of the body tilt prolonged. Furthermore, the results of other previous studies (Lackner and Dizio, 1994; Dizio and Lackner, 1995, 2000; Scheidt et al., 2005) suggested the possibility that the positional errors and variability might be improved based on proprioceptive feedback, such as muscle spindles and joint and skin receptors (for review, Proske and Gandevia, 2009) through the repetition of reaching movements even without visual and tactile feedback about the location of subject’s hand and a target. To evaluate the sustain tilt and learning effects on reaching performance, we first compared the constant errors and intra-subject variability of IDE and FDE with the mean of the initial and last 5 trials by means of a two-way analysis of variance [ANOVA, 5 conditions (0°, ±8°, ±16°) × 2 trial phases (initial and last)]. Subsequently, the effect of body tilt on reaching performance was evaluated by the one-way ANOVA in constant error and intra-subject variability of IDE and FDE. Bonferroni tests were used for *post hoc* comparisons. The significance level for all comparisons was set to 0.05. All statistical analyses were performed with SPSS (IBM, Japan).

### Results

Successful data were obtained from all 14 subjects tested, and were analyzed. The data obtained are summarized in **Table 1**.

**Table 1:** Constant error and intra-subject variability of IDE and FDE at each tilt position.

		Left side down			Right side down	
		-16°	-8°	0°	8°	16°
Constant error	IDE	-1.8 ± 3.2	0.4 ± 2.7	2.9 ± 3.2	4.5 ± 2.2	2.5 ± 2.3
	FDE	-1.8 ± 0.9	-1.6 ± 0.9	-0.3 ± 0.8	1.5 ± 1.0	1.0 ± 0.9
Intra-subject variability	IDE	9.4 ± 0.7	8.6 ± 0.7	6.6 ± 0.6	8.4 ± 1.0	7.8 ± 0.6
	FDE	2.0 ± 0.2	1.7 ± 0.1	1.5 ± 0.1	1.9 ± 0.1	1.8 ± 0.1

*Mean and SE across subjects.*

First, the effect of repetition due to the reaching movement at the sustained tilt condition was examined by comparing the mean of the initial and final 5 trials for constant errors and intra-subject variability of IDE and FDE. For constant errors of IDE, the ANOVA did not show significant main effect either of body tilt [*F*(4,52) = 2.03, *p* = 0.10] or of trial phase [*F*(1,13) = 0.01, *p* = 0.93] with no significant interaction [*F*(4,52) = 2.10, *p* = 0.14]. For those of FDE, two-way ANOVA showed a significant main effect of the body tilt [*F*(4,52) = 7.69, *p* < 0.01], but no significant main effect of trial phase [*F*(1,13) = 1.71, *p* = 0.21] with no interaction [*F*(4,52) = 1.30, *p* = 0.29]. In addition, for intra-subject variability (SD) of IDE, the ANOVA showed a significant main effect of body tilt [*F*(4,52) = 4.76, *p* < 0.01], but no significant main effect of trial phase [*F*(4,52) = 1.12, *p* = 0.31] with no interaction [*F*(4,52) = 1.25, *p* = 0.30]. For that of FDE, the ANOVA showed a significant main effect of body tilt [*F*(4,52) = 4.38, *p* < 0.01], but no significant main effect of trial phase [*F*(1,13) = 0.29, *p* = 0.60] with no interaction [*F*(4,52) = 1.69, *p* = 0.17]. These results indicated that no clear effects of sustained whole-body tilt and motor learning were observed in our experimental setup. Therefore, we omitted the effect of sustained body tilt and pooled all of the collected data through task repetition in a certain tilt, and all of them were equally used for calculating the mean and standard deviation at each condition.

#### Effects of Body Tilt on Accuracy of Reaching Performance

**FIGURE 2:**
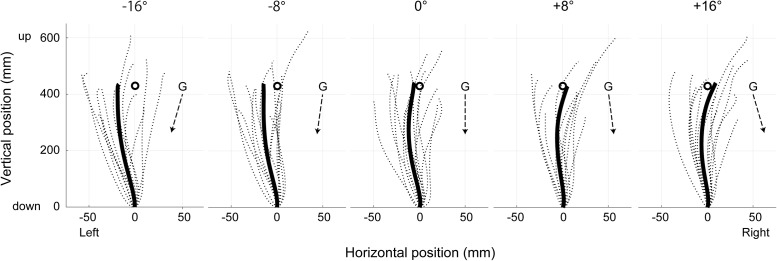
Two-dimensional trajectories of the right fingertip in the egocentric coordinates at each tilt position in roll plane. The dotted lines represent mean trajectories in each subject, whereas solid lines show mean trajectories across subjects. Broken arrow lines represent the gravitational directions in the egocentric coordinates at the each position. Circles denote the location of the visual target presented at subject’s individual eye level.

**FIGURE 3:**
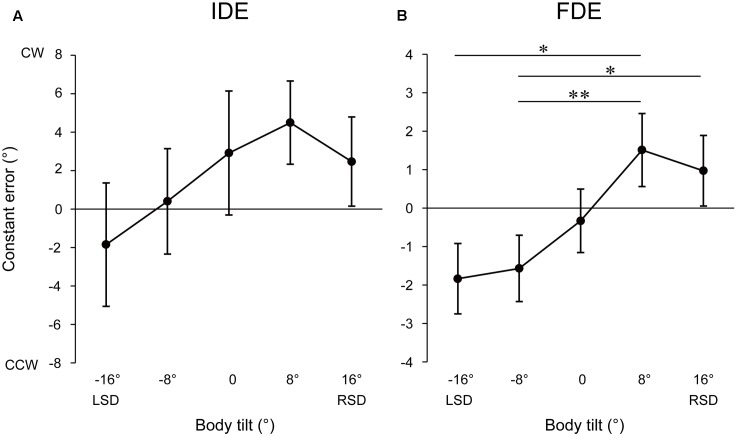
Mean constant errors of IDE **(A)** and FDE **(B)** at each tilt position. Error bars denote standard errors. ^∗^*p* < 0.05; ^∗∗^*p* < 0.01.

**Figure 2** illustrates the mean upper limb trajectories at each tilt position. The trajectories tended to be curved slightly leftward in roll plane, and to terminate to the left of the target when the body was tilted leftward, whereas to terminate to the right of the target when tilted rightward. **Figure 3** shows the mean and standard error (SE) of the constant error in relation to the five-tilt conditions observed for IDE and FDE. For IDE (**Figure 3A**), one-way ANOVA revealed a tendency for the IDE to shift in the direction of body tilt, but the main effect of the body tilt was not statistically significant [*F*(4,52) = 2.55, *p* = 0.08, *r*^2^= 0.16]. In contrast, for FDE (**Figure 3B**), ANOVA revealed a significant main effect of body tilt [*F*(4,52) = 7.69, *p* < 0.001, *r*^2^= 0.37]. Further analysis by the *post hoc* tests elucidated that FDE at -8° was significantly smaller than that at 8° (*p* < 0.01) and 16° (*p* < 0.05). In addition, FDE at -16° was significantly smaller than that at 8° (*p* < 0.05). The effect sizes (η^2^) in ANOVAs for IDE and FDE were 0.31 and 0.71, respectively.

The inter-subject correlation analysis revealed that the correlation between the angle of body tilt and constant errors of IDE was not significant [*F*(1,68) = 2.18, *p* = 0.14, *r*^2^= 0.03]. In contrast, the correlation between the angle of body tilt and constant errors of FDE [*F*(1,68) = 9.64, *p* < 0.01, *r*^2^= 0.12] was significantly positive, indicating that the terminal location of fingertip shifted more largely to the direction of body tilt as the magnitude of the angle of body tilt increased.

In addition, we checked whether or not there was laterality in the effects of body tilt on the IDE and FDE by means of the following method. Firstly, we calculated the effects of body tilt on IDE and FDE by subtracting those at 0° position from those at each tilted position for each subject. Then, we replaced plus and minus signs at the leftward tilted position (-8° and -16°) to produce a new dataset of tilt effects, in which plus and minus correspond to the deviation toward the tilted side and that toward opposite side to body tilt. Finally, we conducted two-way repeated-measure ANOVAs (2 tilt sides × 2 tilt angles) on this dataset. The results revealed that the main effects of tilt side were not significant for both IDE [*F*(1,13) = 0.42, *p* = 0.53] and FDE [*F*(1,13) = 0.02, *p* = 0.88] with no significant interactions between tilt side and tilt angle [IDE: *F*(1,13) = 0.18, *p* = 0.68, FDE: *F*(1,13) = 0.12, *p* = 0.74], indicating that there was no effective laterality in the effects of body tilt on the performance of upper limb reaching movements.

#### Effect of Body Tilt on the Precision of Reaching Performance

The precision of the reaching performance was evaluated by observing the standard deviation of the IDE and FDE for each subject (**Table 1**). ANOVA indicated the significant main effect of the body tilt on intra-subject variability both for IDE [*F*(4,52) = 5.57, *p* < 0.05] and for FDE [*F*(4,52) = 2.90, *p* < 0.05]. The *post hoc* tests showed that IDE for -16° and -8° were significantly greater than for 0° (both at *p* < 0.05), whereas no significant differences were found for any pair of tilt conditions in FDE.

### Discussion

The purpose of this study was to investigate how the performance of upper limb movements was modulated at a small angle of whole-body tilt in roll plane. We examined spatial properties of upper limb reaching movements toward a memorized target during whole-body tilt in roll plane. We hypothesized that upper limb movements are shifted toward the direction opposite to body tilt owing to overestimation of the body tilt. In contrast to our expectations, the result showed that the terminal location of the fingertip during the memory-guided reaching task tended to shift to the direction of the body tilt away from the target location, which rejected our hypothesis.

The CNS internally represents the surrounding space based on reference frames defined either egocentrically, by referring one’s body parts such as eye, head, or trunk; or allocentrically, by referring spatial cues in the surrounding environment (for review, Desmurget et al., 1998). Since the CNS is considered to be responsible for planning and commanding body movements based on these reference frames, the accuracy of the internal representation of these reference frames is critical for acquiring good quality of motor performance. In a situation where none of the allocentric cues are available from the surrounding environment, one has to depend only on the egocentric cues, which in turn indicates that motor performances are planned and commanded solely depending on the egocentric reference frame regardless of the spatial relationship between the body and the surrounding environment.

Previous studies showed that when the egocentric coordinate was spatially shifted in roll plane and was dissociated from the gravitational coordinate, perception of the body longitudinal axis, representing the egocentric reference frame, suffered an error (Bauermeister, 1964; Ceyte et al., 2007, 2009). In those studies, the perceived direction of the body longitudinal axis was further tilted toward the direction of the body tilt from the true direction during whole-body roll tilt. These facts led us to the alternative interpretation, instead of the preceding hypotheses, that the inaccuracy of upper limb movements during whole-body tilt in roll plane was attributed to the perceived direction of the body longitudinal axis rather than that of gravity. A conceivable interpretation of the internal mechanism is that sensory stimulation, such as that of proprioceptive and vestibular organs, induced by whole-body tilt would have disturbed the establishment of the internal egocentric representation of the body in space for planning movements in which the dorsal visual pathway and posterior parietal cortex (PPC) play a major role (Stein, 1989; Goodale and Milner, 1992). As a result, the motor command produced based on the internal egocentric representation suffered errors in space, as was seen in the shift of the terminal location in the current study. In spite of the alternative hypothesis, we had no idea if the perceived direction of the body longitudinal axis is actually shifted in space under the current experimental setup. To obtain concrete evidence, we further investigated properties of the internally represented egocentric reference frame during whole-body roll tilt in the following experiment.

## Experiment 2

In this experiment, we tried to elucidate the relationship between the motor control of the upper limb and internal representation of space when the body was tilted in roll plane. We examined if the previously known tendency for the perceived direction of the body longitudinal axis to shift to the direction of body tilt relative to the true direction in roll plane (Bauermeister, 1964; Ceyte et al., 2007, 2009) would appear in our experimental setup, and directly related the performance of the spatial perception to motor behavior observed in the Experiment 1.

### Methods and Materials

#### Subjects

Seven healthy male subjects (subject ID 1, 4, 5, 8, 9, 11, and 13 in Experiment 1) were assigned for Experiment 2 on a different day from that of Experiment 1.

#### Apparatus

Similar to Experiment 1, subjects sat on the seat mounted on the tilt table in a darkened room, and their head, trunk, and legs were restrained to the seat with belts. A liquid crystal display (LCD, LTN097QL01, SAMSUNG, Korea) sized 19.6 cm (height) × 14.7 cm (width) was placed 20.0 cm in front of the subject’s head. Between the subject’s head and the display, a dark-colored cylinder wherein one end was covered by a dark-colored board with a hole (13-cm diameter) on the center was placed to avoid the display edge and other spatial cues slipping in. A luminous line (of 7.5-cm length, and 0.1-cm width) appeared on the center of the display, which could be rotated around the midpoint of the line by the subject’s manipulation of the game controller (BSGP1204, iBUFFALO, Japan). During the experiment, the subjects were provided with white noise via the earphones to prevent any spatial cues due to the noise arising from the surrounding environment.

#### Procedure

First, the tilt table was slowly tilted right- or leftward in roll plane from the upright position at 2.75°/s. Similar to Experiment 1, five conditions of the whole-body tilt in roll plane (0°, ±8°, ±16°) were applied. Five hundred milliseconds after the tilt position was achieved, a single beep prompted the subjects to start adjusting the luminous line to the direction of their body longitudinal axis. The initial direction of the luminous line was randomized at ±45°, ±60°, or 90° from the direction of the body longitudinal axis. The subjects successively repeated 10 trials at the identical position, which consisted one block, and then the subject was returned to the initial upright position. Each subject performed five blocks, with a total of 50 trials. The first block was executed with the upright (0°) condition, and then the other tilt conditions were followed by a randomized order.

#### Data Analysis

The subjective direction of the body longitudinal axis obtained by the adjustment of the luminous line, i.e., subjective body orientation (SBO), was recorded, and its angular dissociation from the objective value of the body tilt was calculated for each trial. Positive and negative values correspond to the clockwise (rightward) and counterclockwise (leftward) direction rotation in roll plane from the subject’s view, respectively. The effect of the body tilt on SBO was evaluated using one-way ANOVA. Furthermore, the correlations between constant errors of SBO and those of IDE or FDE in the reaching task observed in Experiment 1 at each tilt position were evaluated using stepwise multiple regression analyses in which FDE as an objective variable, and SBO and the body tilt angle as explanatory variables were set.

### Results

**FIGURE 4:**
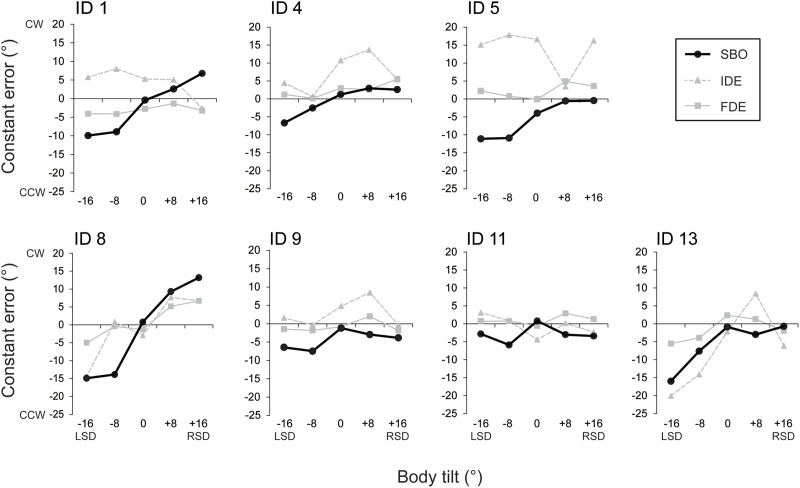
Constant errors of SBO at each tilt position (filled circles with solid line). Data from each subject are separately shown. The constant errors of IDE (filled triangles and gray broken line) and those of FDE (filled squares and gray solid line) are also depicted in the figures for comparison.

**FIGURE 5:**
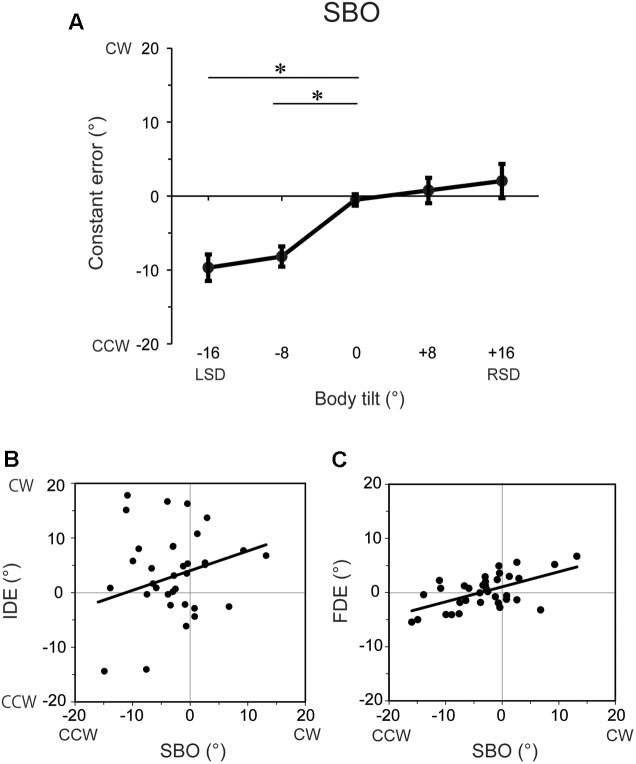
**(A)** Constant errors of SBO at each tilt position averaged across subjects. Error bars denote standard errors across subjects. ^∗^*p* < 0.05. **(B)** Correlation between constant errors of SBO and those of IDE. **(C)** Correlation between constant errors of SBO and those of FDE. Each filled circle represents the mean constant errors for each subject at each tilt position and solid lines indicate the single regression of the data points.

The constant errors of SBO at each tilt position for each subject are shown in **Figure 4**. IDE and FDE were also inserted in the figure for comparison. The inter-subject results of the constant errors of SBO are summarized in **Figure 5A**. One-way ANOVA revealed a significant main effect of body tilt on constant errors of SBO [*F*(4,24) = 10.8, *p* < 0.01, η^2^= 0.46]. *Post hoc* tests elucidated that constant errors of SBO at 0° were significantly larger than at -8° and -16° (both at *p* < 0.05). The single-regression analysis revealed that the body-tilt angle and constant errors of SBO were significantly positively correlated [*F*(1,33) = 36.2, *r*^2^= 0.52, *p* < 0.001].

The relationship between constant errors of SBO and those of IDE or FDE observed in Experiment 1 are shown in **Figures 5B,C**. For IDE, the multiple regression analysis revealed that no factors were included in the regression model, indicating that SBO and IDE were not significantly correlated. In contrast, for FDE, the multiple regression analysis revealed that SBO was the only factor included in the regression model, and constant errors of FDE and SBO were significantly correlated (β = 0.57, *p* < 0.001, *r*^2^= 0.34) with large effect size (cohen’s *f*^2^= 0.50). This result indicates that terminal locations of the fingertip were shifted to the perceived direction of body longitudinal axis.

### Discussion

We observed that the perceived direction of the body longitudinal axis was tilted toward the direction of body tilt in roll plane as reported in the previous studies (Bauermeister, 1964; Ceyte et al., 2007, 2009). This result supports our hypothesis that the perceived body longitudinal axis shifted to the direction of body tilt relative to the true direction tilt; in other words, the internally represented egocentric reference is tilted due to the whole-body tilt in roll plane.

## General Discussion

The purpose of this study was to investigate how the motor performance of goal-directed upper limb movements would be affected when the gravitational direction relative to the body orientation was changed by whole-body tilt in roll plane (Experiment 1). Furthermore, we investigated if the properties of the resultant motor performances in Experiment 1 were attributed to the shift of the perceived egocentric space, represented by the perceived direction of body longitudinal axis (Experiment 2). Results showed that the terminal location of the fingertip shifted to the direction of body tilt (Experiment 1), and the direction of its shift was effectively related to the perceived direction of body longitudinal axis (Experiment 2). These results suggest that the motor planning of upper limb movements during whole-body roll tilt without allocentric visual cues depends largely on internally represented egocentric reference frame. Consequently, the upper limb movements would have shifted along the perceived direction of body longitudinal axis away from the actual target.

Previously, Prieur et al. (2006) studied motor performances of the reaching task toward the memorized target and demonstrated that the accuracy was maintained even when the whole body was tilted in roll plane by 17.5°. This result contradicts that of our current study which showed a shift of the terminal location toward the direction of the body tilt. A conceivable interpretation could be induced by the difference in the plane where reaching movements were performed. In the study of Prieur et al. (2006), the reaching task was performed in a horizontal plane, while it was done on roll plane in this study. It was demonstrated that whole-body tilt in roll plane shifts the perceived direction of body longitudinal axis in roll plane, but not in the pitch plane (Ceyte et al., 2007), suggesting that shift of the egocentric reference frame due to whole-body tilt depends on the corresponding plane of body tilt. Therefore, it is assumed that whole-body tilt in roll plane did not induce a shift of egocentric reference frame on the horizontal plane, resulting in the accuracy of the memory-guided reaching task not being affected in horizontal plane in the study by Prieur et al. (2006). From another perspective, it is assumed that the mechanical force applied to the upper limb was similar in the study by Prieur et al. (2006). In both studies, as the body tilted, the gravitational component slipped into a horizontal direction, which would have pulled the upper limb laterally. Therefore, the mechanical feature cannot explain the discrepancy between motor performances of the reaching task in the study by Prieur et al. (2006) and the current study. Accordingly, the shift in upper limb movement observed in the present study would be attributed to the shift of the perceived direction of body longitudinal axis.

The influence of whole-body tilt on reaching performance was separately analyzed and evaluated in its initial part (IDE) and final part (FDE) since these were considered to be produced by different internal mechanisms, majorly reflecting the motor planning process in the initial part whereas an online-control process in addition to the motor planning process was reflected in the final part. The results of this study indicated that whole-body tilt more strongly influenced FDE than IDE. In addition, SBO, representing perceived body orientation was correlated better in FDE than in IDE. Therefore, it is suggested that the final part, instead of the initial part, was more affected by whole-body tilt in roll plane. The comprehension of this phenomenon probably requires some internal mechanisms processing motor outputs. A conceivable factor is the “signal-dependent noise (SDN)” denoting the noise that accompanies the production of the motor command according to its magnitude (Harris and Wolpert, 1998; Jones et al., 2002). Muscle activities of the upper limb at the initiation of rapid upward movements against gravity are known to largely suffer from SDN (Papaxanthis et al., 2003). Given this fact, it is possible that the emergence of SDN was greater in the initial part of upper limb movements than in the final part, resulting in the clear effects of the whole-body tilt on FDE while not on IDE. In fact, intra-subject variability of IDE was much larger than that of FDE (see the Results in Experiment 1).

Beyond our consideration, we should note a limitation that the effect of whole-body tilt on the upper limb movements was tested only under relatively small angles of body tilt, such as 8° and 16°. The performance of upper limb reaching during whole-body tilt possibly depends on the body tilt amplitude relative to gravity. Bourdin et al. (2001) found that the pointing accuracy was maintained during forward or backward body tilt at small angles, such as 2°, 4°, or 8° in the pitch plane, whereas Smetanin and Popov (1997) showed that the terminal locations of upper limb movements were deviated more upward during large body tilts, such as supine or prone position compared with upright position. These facts suggest that the results obtained in the present study could not necessarily be applied to the situation where the body is tilted at a larger angle in roll plane. Therefore, further experiments are required to test the effect of body tilt on motor performance of goal-directed movements by using large body tilts in roll plane.

## Conclusion

The present study demonstrated that the dissociation between the egocentric and gravitational coordinates induced by whole-body tilt leads to the systematic shifts of the egocentric reference frame for action, which, in turn, influences goal-directed upper limb movements with no allocentric visual cues available. These results suggest that we can accurately and stably perform aimed goal-directed upper limb movements even during small changes of body orientation relative to gravity because of the CNS compensation for this effect of whole-body tilt using visual information about body and surrounding environment.

## Author Contributions

KT developed the study concepts, designed and performed the experiments, analyzed the data, and wrote the paper. YS, SY, and YK assisted in designing experiments and writing the paper. KK designed the experiments and wrote the paper.

## Conflict of Interest Statement

The authors declare that the research was conducted in the absence of any commercial or financial relationships that could be construed as a potential conflict of interest.
